# Nutritional status mediates the relationship between depression and mild cognitive impairment among Chinese community-dwelling older adults

**DOI:** 10.3389/fpsyt.2026.1684063

**Published:** 2026-03-04

**Authors:** Xiaoyu Chen, Peipei Han, Zhenwen Liang, Liou Cao, Jing Gao, Qi Guo

**Affiliations:** 1 Department of Rehabilitation Medicine, Shanghai University of Medicine and Health Sciences Affiliated Zhoupu Hospital, Shanghai, China; 2 Department of Nephrology, Molecular Cell Lab for Kidney Disease, Ren Ji Hospital, Shanghai Jiao Tong University School of Medicine, Shanghai, China; 3 General Practice Clinic, Pujiang Community Health Service Center in Minhang District, Shanghai, China

**Keywords:** depression, mediatation, mild cognitive impairment, nutritional status, older adults

## Abstract

**Background:**

Mild cognitive impairment (MCI) and depression are prominent public health concerns among older adults, closely linked to their nutritional status. Our study aimed to explore the association and mediation pathways involving depression, nutritional status, and MCI in this population.

**Methods:**

The study included 4799 community-dwelling Chinese older adults aged 60 years and older in Tianjin and Shanghai, China. We utilized the 30-item Geriatric Depression Scale (GDS-30) to assess depression presence and severity. A GDS-30 score ≥11 indicated the presence of depression. Participants were categorized by symptom severity: “None” (GDS-30<11), “Mild” (11≤GDS-30 ≤ 20), “Moderate to severe” (20<GDS-30 ≤ 30). Nutritional status was evaluated using the Mini Nutritional Assessment-Short Form (MNA-SF). Cognitive function was assessed with the Mini-Mental State Examination (MMSE), and daily living activities were measured using the Instrumental Activities of Daily Living (IADL). Logistic regression and mediation analyses were conducted with full adjustment for potential confounding factors.

**Results:**

Among 4799 older adults (2010 men, mean age 71.5 ± 5.8 years), 11.8% exhibited depression, and 12.1% had MCI, while 18.8% were at risk of malnutrition, and 1% were malnourished. Participants at risk of malnutrition or experiencing depression were associated with MCI. Additionally, nutritional status significantly mediated the relationship between depression and cognitive function, with a slightly larger mediating effect, particularly in cases of mild depression.

**Conclusions:**

Individuals at risk of malnutrition or depressed were associated with MCI. Poor nutritional condition mediates the association between depression and MCI in older adults. Early nutritional interventions may mitigate cognitive decline in depressed older adults.

## Introduction

As the global population of older adults continue to grow, cognitive impairment has emerged as a major risk factor for poor health and a significant public health burden. Recent studies estimate that over 15 million people aged 60 or older in China have dementia (1). Mild cognitive impairment (MCI) represents an intermediate state between normal aging and dementia, with approximately 10% of individuals with MCI progressing to dementia annually, compared to a 1-3% conversion rate in the general older adult population (2, 3). Given its high prevalence, rapid progression to dementia, and substantial healthcare costs, MCI has become a pressing public health challenge.

Depression is a prevalent mental disorder among older adults and a leading cause of emotional distress in later life (4). Regrettably, depression often goes undiagnosed or untreated in this population (5). Growing evidence suggests that depression independently accelerates the progression from MCI to dementia (6, 7). However, the relationship between depression and MCI is complex, and a vicious cycle may exist between the two conditions (8). Thus, depression deserves increased attention in the care of individuals with MCI. It is worth noting that malnutrition is another critical health issue for older adults, particularly as the aging population grows (9, 10). It affects older adults psychologically through mechanisms such as brain atrophy and reduced enjoyment of food (11, 12). Depression has been proposed as a risk factor for malnutrition in this population (13, 14). Additionally, poor nutritional status plays a role in the development and progression of cognitive decline (12, 15, 16), effective dietary approaches to enhance cognitive function (17). While some studies have explored the relationship between depression, malnutrition, and cognition (18, 19), their focus on total cognitive impairment (including patients with dementia) rather than specifically on MCI. The interrelationship of these three conditions in old adults remains unclear.

Therefore, this study aims to examine the relationship between depression, nutritional status and MCI in community-dwelling individuals aged 60 years or older. We explore the possible mediating effects of nutritional status on the association between depression and cognitive function. Additionally, we test whether nutritional status mediates the relationship between different severity levels of depression and cognition.

## Methods

### Participants

The study population comprises residents aged 60 years or older from Tianjin and Shanghai, China, who participated in China’s National Free Physical Examination program between August 2018 and October 2022. Participants were excluded as following criteria: (1) did not complete date for depression, nutrition assessments and cognitive function; (2) had a known diagnosis of dementia; (3) unable to communicate with the study staff or provide informed consent. After excluding 306 subjects, the final sample consisted of 4799 participants (2010 males, 2789 females). Missing data on cognitive function assessment, psychological tests, and nutritional status evaluation were noted in 186, 25, and 7 participants, respectively. Another 6 participants had missing data on basic covariates, and 82 were evaluated for dementia. The study received ethical approval from the Ethics Committee of Tianjin Medical University and Shanghai Medical and Health University, adhering to the Declaration of Helsinki.

### Covariates

Interviews were conducted using questionnaires from previous studies (20). Socio-demographic variables included age, sex, marital status, living arrangements, and education. Behavioral characteristics such as alcohol consumption, smoking, physical activity, and falls history were recorded. Chronic diseases, including diabetes mellitus, hypertension, hyperlipidemia, coronary heart disease, stroke, kidney disease, peptic ulcer, biliary tract disease, pulmonary disease, osteoarthritis, Parkinson’s disease, gout, cancer, and thyroid disease, were also documented (21).

### Definition of MCI

MCI was defined according to Petersen’s diagnostic criteria (22). Mini-Mental State Examination (MMSE) and Instrumental Activities of Daily Living scale (IADL) were used. Cognitive impairment thresholds for MMSE were ≤17, 20, and 24 points for illiteracy, elementary, and middle school or above, respectively (23). IADL consists of eight items on a scale of 0 to 8, with higher scores indicating better daily living ability. IADL score ≥6 indicates normal daily living ability (24, 25).

### Assessment of depression

Depression was screened by the Chinese version of 30-item Geriatric Depression Scale (GDS-30), which was standardized including 30 items ranging from 0–30 points. A cutoff value of 11 points was used to define depression (26). Participants were also grouped according to the severity of symptoms: “None” (GDS-30<11), “Mild” (11≤GDS-30 ≤ 20), “Moderate to severe” (20<GDS-30 ≤ 30).

### Nutritional status evaluation

Nutritional status was evaluated using the Mini Nutritional Assessment-Short Form (MNA-SF), a validated screening tool used in geriatric health care (27). Compared with the MNA, the sensitivity and specificity of this version are 97.9% and 100%, respectively (28). The MNA-SF consists of six items assessing food intake, weight loss, mobility, psychological stress, neuropsychological problems, and body mass index (BMI). The total MNA-SF score ranges from 0 to 14, with the score of 12-14, 8-11, and 0–7 defined as being well-nourished, being at risk of malnutrition, and malnourished, respectively.

### Statistical analyses

Statistical analyses were performed using SPSS version 26.0 (IBM Corp., Armonk, NY, USA). Continuous variables were expressed as means ± standard deviations (SD), while categorical variables were expressed as frequencies and percentages. Group differences were analyzed using the independent t-test or chi-square test, as appropriate. Univariate logistic regression analysis was conducted to explore the association between depression, nutritional status, and MCI. Multivariate logistic regression models were then used to adjust for potential confounding factors.

Mediation analyses were performed to explore the mediating role of nutritional status in the association between depression and MCI. In the mediation analysis model with full adjustment, we included GDS score and depression severe grades as independent variables (X), MMSE score as dependent variable (Y), and the MNA-SF variable as a potential mediator (M). The total effect (path c) represents the sum of the direct and indirect effects of GDS score and depression severe grades on MMSE score. The direct effect (path c’) is the effect of GDS score and depression severe grades on MMSE, and the indirect effect (path ab) is the mediating effect of the association between GDS score and depression severe grades and MMSE.

The PROCESS macro for SPSS was used for mediation analysis (29). We tested the significance of the indirect effect (mediation effect) and confirmed that the effect of independent variable on mediator, the effect of mediator on dependent variable and the total effect of independent variable on dependent variable were significant, respectively. A bootstrapping method with 5000 resamples was applied to calculate bias-corrected 95% confidence intervals for direct and indirect effects.

## Results

### Participant characteristics

Among 4799 participants (2010 men, 2789 women) who were available to be analyzed, 583 (12.1%) had MCI, in which 9.4% for men and 14.2% for women respectively. **Table 1** presents the socio-economic and health-related characteristics of individuals stratified by cognitive state. We found that participants with MCI tended to be older, female, widowed, living alone, having low education levels (P<0.05, **Table 1**). In regard to the health-related variables, falling, depression, poor nutrition, hyperlipidemia, coronary heart disease stroke, peptic ulcer and osteoarthritis were significantly related to MCI (P<0.05, **Table 1**). Regarding nutritional status, 18.8% (902 participants) were at risk of malnutrition (MNA-SF score 8-11), and 1% (50 participants) were malnourished (MNA-SF score 0-7).

**Table 1:** Characteristics of study participants according to the status of cognition.

Characteristic	Normal	MCI	P Value
	(n=4216)	(n=583)	
Age(y)	71.27 ± 5.56	73.27 ± 6.91	<0.001
Sex			<0.001
Male (%)	1822(43.2)	188(32.2)	
Female (%)	2394(56.8)	395(67.8)	
BMI (kg/m²)	23.96 ± 3.31	24.08 ± 3.51	0.394
Widowed (%)	618(14.7)	149(25.6)	<0.001
Living alone (%)	516(12.2)	125(21.4)	<0.001
Education level (%)			<0.001
Less than high school	1972(46.8)	370(63.5)	
High school or higher education	2244(53.2)	213(36.5)	
Drinking (%)	401(9.5)	59(10.1)	0.062
Smoking (%)	625(14.8)	91(15.6)	0.065
Economic status (%)			<0.001
<1000 RMB/mo	206(4.9)	97(16.6)	
1000–3000 RMB/mo	1305(31.0)	260(44.6)	
3000–5000 RMB/mo	818(19.4)	83(14.2)	
≥5000 RMB/mo	1887(44.,8)	143(24.5)	
IPAQ (Met-min/wk)	3759(1386,6720)	3360(1386,6426)	0.062
Fall history (%)	313(7.4)	71(12.2)	<0.001
GDS score	5.29 ± 4.47	6.86 ± 5.69	<0.001
Depression (%)	459(10.9)	109(18.7)	<0.001
Severity of depression (%)			<0.001
None	3757(89.1)	474(81.3)	
Mild	400(9.5)	87(14.9)	
Moderate to severe	59(1.4)	22(3.8)	
MNA-SF	12.69 ± 1.61	12.37 ± 1.69	<0.001
Nutritional status (%)			0.003
Well nourished	3410(80.9)	437(75.0)	
At risk of malnutrition	764(18.1)	138(23.7)	
Malnourished	42(1.0)	8(1.4)	
Chronic conditions (%)			
Diabetes mellitus	828(19.6)	115(19.7)	0.961
Hypertension	2881(68.3)	423(72.6)	0.039
Hyperlipidemia	2363(56.0)	290(49.7)	0.004
Coronary heart disease	1002(23.8)	168(28.8)	0.008
Stroke	401(9.5)	77(13.2)	0.005
Kidney disease	269(6.4)	32(5.5)	0.405
Biliary tract disease	579(13.7)	68(11.7)	0.170
Peptic ulcer	406(9.6)	39(6.7)	0.022
Pulmonary disease	310(7.4)	37(6.3)	0.379
Osteoarthritis	471(11.2)	85(14.6)	0.016
Parkinson disease	29(0.7)	6(1.0)	0.364
Gout	203(4.8)	15(2.6)	0.015
Cancer	140(3.3)	15(2.6)	0.338
Thyroid disease	229(5.4)	25(4.3)	0.248

BMI, body mass index; MNA-SF, Mini Nutritional Assessment-Short Form; IPAQ, international physical activity questionnaire; Met-min/wk, metabolic equivalent task minutes per week; GDS, Geriatric Depression Scale.

### Association Between Depression, Nutritional Status, and MCI

Logistic regression analysis revealed significant associations between depression, nutritional status, and MCI (**Table 2**). After adjustments for potential confounders (age, sex, BMI, widowed, living alone, education, fall history, hypertension, hyperlipidemia, coronary heart disease, stroke, peptic ulcer, osteoarthritis and gout), we observed that total MNA-SF score (OR = 0.87, 95% CI = 0.82–0.92) was significantly associated with MCI. We also found that older adults at risk of malnutrition (MNA-SF score 8-11) (OR = 1.44, 95% CI = 1.14–1.81) were significantly associated with MCI compared to those with well nutritional status (MNA-SF score 12-14) stratified by nutritional status. Furthermore, depression (OR = 1.56, 95% CI = 1.22–1.99) was significantly related to MCI. Interestingly, graded by depression severity, the association with MCI increased as the severity of depression increased (Mild depression: OR = 1.48, 95% CI = 1.14–1.93; Moderate to severe depression: OR = 2.02, 95% CI = 1.14–1.93).

**Table 2:** Associations of MCI with nutritional status and depression in a logistic regression analysis.

Variables	OR (95%CI)			
	Crude	*P*	Adjusted model	*P*
MNA-SF score	0.89(0.85,0.94)	<0.001	0.87(0.82,0.92)	<0.001
Nutritional status				
Well nourished	Ref		Ref	
At risk of malnutrition	1.41(1.15,1.73)	0.001	1.44(1.14,1.81)	0.002
Malnourished	1.49(0.69,3.19)	0.308	1.74(0.78,3.88)	0.173
Depression	1.88(1.50,2.37)	<0.001	1.56(1.22,1.99)	<0.001
Depression severe grades				
None	Ref		Ref	
Mild	1.72(1.34,2.22)	<0.001	1.48(1.14,1.93)	0.003
Moderate to severe	2.96(1.80,4.87)	<0.001	2.02(1.18,3.44)	0.010

Adjusted model: Adjusted for age, sex, BMI, widowed, living alone, education, fall history, hypertension, hyperlipidemia, coronary heart disease, stroke, peptic ulcer, osteoarthritis, gout, MNA-SF score or depression.

### Mediation analysis

**Figure 1:**
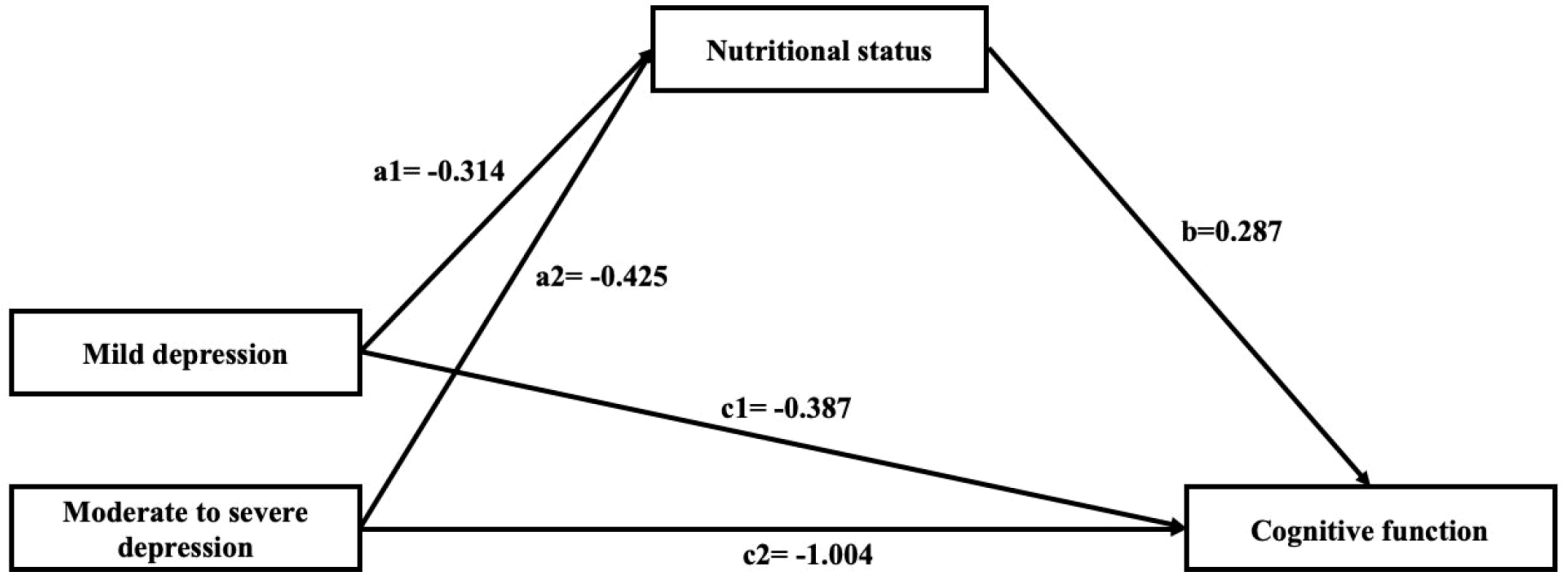
Multi-categorical mediation model of depression, nutritional status, and cognitive function in suburb-dwelling Chinese older adults. Paths a1 and a2 represent the effect of mild depression and moderate to severe depression on nutritional status; path b represents the effect of nutritional status on cognitive function; paths c1 and c2 represent the relative direct effect of mild depression and moderate to severe depression on cognitive function. Unstandardized regression coefficients are reported. This model represents a hypothesized association pathway rather than establishing causal directionality.

Mediation analyses were conducted to explore the potential mediating role of nutritional status in the association between depression and MCI. **Table 3**, **Figure 1** showed that MNA-SF score had a significant mediating role in the association between depression and MCI. Poor nutritional status plays a mediate role in the association between mild depression (indirect effect ab = -0.090; 95%CI= -0.149 to -0.046), and moderate to severe depression (indirect effect ab = -0.122; 95%CI= -0.270 to -0.004) with cognitive function, and the mediating effect was enhanced slightly larger in mild depression (18.9%) than moderate to severe depression (10.8%).

**Table 3:** Summary of mediation analysis for depression, MNA-SF and MMSE score.

Independent variable	Mediating variable	Dependent variable	Coefficient (bias-corrected bootstrap 95% CI)			Relative Proportion	
Depression	Nutritional status	cognition function	Indirect effect (ab)	Total effect (c)	Direct effect (c’)	ab/c	c’/c
GDS	MNA-SF	MMSE score	-0.010(-0.014, -0.006)	-0.072(-0.094, -0.050)	-0.062(-0.084, -0.040)	13.9%	86.1%
Depression severe grades							
None			Ref	Ref	Ref		
Mild			-0.090(-0.149, -0.046)	-0.477(-0.812, -0.142)	-0.387(-0.721, -0.054)	18.9%	81.1%
Moderate to severe			-0.122(-0.270, -0.004)	-1.126(-1.957.-0.296)	-1.004(-1.830, -0.178)	10.8%	89.2%

MNA-SF, Mini Nutritional Assessment-Short Form; GDS, Geriatric Depression Scale; MMSE, Mini-Mental Status Examination.

## Discussion

The present study used mediation models to explore the mediation role of nutritional status between two important clinical conditions: depression and cognitive impairment. Our results suggest that individuals at risk of malnutrition and depression were both significantly associated with MCI in older community-dwelling Chinese adults. Notably, moderate to severe depression exhibits a stronger link with MCI compared to mild depression. Furthermore, our study reveals that poor nutritional status significantly mediates the association between depression and cognitive function, with the mediating effect being particularly pronounced in cases of mild depression.

In our investigation, the prevalence of MCI was 12.1%, consistent with recent Chinese national study involving 46011 adults aged 60 years or older (15.5%) (1). A systematic review of 22 Chinese studies in older adults also yielded similar results (12.7%) (30). The prevalence of depression using the GDS-30 was 11.8%, aligning with our prior studies (21, 25). Regarding nutritional status, 1% of the older adults were malnourished, 18.8% were at risk of malnutrition, and 80.2% were well-nourished. Our results are line with seven previous studies involving 2798 community- dwelling elderly persons, reporting that 1% were malnourished, 29% were at risk of malnutrition, and 70% were well-nourished (31). However, variations exist; for instance, Mantzorou et al. (18) reported 11.3% malnourished participants, while another study indicated 20% malnutrition and 49% at risk of malnutrition (31–33). The reason for the difference may be that the nutritional status of older people living in nursing homes, long-term care facilities and hospital settings is poorer than that of older adults who can live independently in the community. In addition, with the welfare policies of the Shanghai government and advanced medical resources, the physical condition of the older adults in the region is generally better than the rest of the country.

Additionally, our results align with previous evidence on the association between nutritional status and cognitive function (15, 18, 34). Accumulating evidence suggest that nutrition is important for optimizing cognition and reducing the risk of dementia (16, 35, 36). While our findings slightly differ, showing that individuals at malnutrition risk (OR = 1.44, 95%CI=1.14,1.81) are associated with MCI, whereas this relationship is absent in malnourished (OR = 1.74, 95%CI=0.78,3.88) older adults, the small prevalence of malnutrition (1%) and limited sample size may explain this lack of association. We anticipate that with a larger sample, the correlation would become statistically significant. Overall, the association between depression and MCI consisted with the results of our previous studies (25). Steffens et al. reported that depression increase the risk for progression from MCI to Alzheimer’s disease dementia (37). Although previous study demonstrated that depression is relation to clinical symptoms onset of MCI, the casual relationship between depression and MCI remains to be explored (38).

Although previous studies have explored the relationship between malnutrition, depression and cognitive function (39), no other studies have highlighted malnutrition as a mediator of the relationship between depression and cognitive function in older adults. Our results suggest that depression is associated with poorer nutritional status, which in turn is associated with lower cognitive performance. This implies that improving nutritional status may mitigate the negative impact of depression on cognitive function. Given the limited effective treatments for depression and cognitive deterioration in older adults, modifiable risk factors, such as nutritional status, offer intervention opportunities. In mediation analysis, the relationship between depression and cognitive function comprises a relative direct effect and a relative indirect effect. When deeper categorization of depression into mild and moderate to severe, both the direct effect (81.1%) and indirect effect (18.9%) of nutritional status remain significant in mild depression, while the direct effect (89.2%) and indirect effect (10.8%) remain significant in moderate to severe depression, albeit to a lesser extent as a mediating effect. This suggests that nutritional status mediates the association between depression and cognitive function, especially concerning mild depression. The total effects of depression on cognitive function increase with depression severity, while the proportion of relative indirect effects (mediated by nutritional status) decreases with depression severity (**Table 3**). The smaller mediation proportion in moderate-severe depression may reflect measurement limitations, smaller subgroup size, or more complex, multi-factorial pathways, and should be explored in longitudinal studies.

Exploration of these mediation pathways may clearly identify underlying associations, which may provide an opportunity for early detection, intervention, and monitoring of the most vulnerable older adults. These findings highlight the importance of integration consideration of psychological health as well as nutritional aspects into clinical practice throughout the lifetime of the older adults to prevent cognitive disorder. We believe that the current research findings indicate that in the management of chronic diseases in communities, greater emphasis must be placed on the screening and intervention of mental health and nutritional status.

Several limitations characterize our study. First, the cross-sectional design limits our ability to establish causality, and only longitudinal studies can unravel the complex and bidirectional association between depression and MCI (37, 38). Future research should extend follow-up times, increase sample sizes, and explore these associations longitudinally. Second, all participants recruited in this present study were relatively healthy, since individuals who were unable to participate in the free annual national physical examination (e.g. those bedridden or diagnosed with serious diseases) were excluded. Consequently, our findings may underestimate the actual prevalence of depression, malnutrition, or MCI. Furthermore, the generalizability of these findings is limited when applied to more vulnerable or institutionalized populations. Thirdly, the MNA-SF scale includes items related to appetite and psychological stress. Given that depressive symptoms can affect appetite and psychosocial functions, there may be conceptual overlap between the MNA-SF and the GDS scales, which could lead to deviations in the estimation of the mediating effect. Further and more in-depth research is needed in the future to explore the relationship between nutrition and depression. Fourthly, although MMSE has certain advantages when applied to large population samples, it has certain limitations in accurately identifying MCI. This may lead to an underestimation of early MCI patients and cause certain classification bias. Fifthly, multi-morbidity may affect the mediating associations observed in this study. Several relevant medical and lifestyle confounders-such as inflammation, polypharmacy, and appetite changes-were not considered, which may bias the estimates. Future work should aim to better clarify the combined effect of these factors under more simulated or controlled conditions.

## Conclusion

In conclusion, our study demonstrated that the association between depression and MCI may be in part mediated by nutritional status. Improving the nutritional status of depressed older adults may counteract the cognitive decline linked to depression. Early nutritional intervention is recommended for improved prevention.

## Data Availability

The raw data supporting the conclusions of this article will be made available by the authors, without undue reservation.
